# Making meaning through cultural embeddedness: Advancing culture‐sensitive training for Alzheimer’s Disease and Alzheimer’s Disease Related Dementia Caregivers in Ibadan, Nigeria

**DOI:** 10.1002/alz.092623

**Published:** 2025-01-09

**Authors:** Isaac Akinkunmi Adedeji, Saheed Akinmayowa Lawal, Adesola Ogunniyi, David C Henderson, Temitope Hannah Farombi, Olufisayo Oluyinka Elugbadebo

**Affiliations:** ^1^ Olabisi Onabanjo University, Ago‐Iwoye, Ogun Nigeria; ^2^ Simon Fraser University, Burnaby, BC Canada; ^3^ Babcock University, Ilisan‐Remo, Ogun Nigeria; ^4^ University of Ibadan, Ibadan Nigeria; ^5^ Boston University, Boston, MA USA; ^6^ University College Hospital, Ibadan Nigeria; ^7^ College of Medicine, University of Ibadan, Ibadan Nigeria

## Abstract

**Background:**

Limited knowledge exists about the cultural approaches to managing the psychological and behavioral outcomes (PBO) of Alzheimer’s Disease and Alzheimer’s Disease‐Related Dementia (AD/ADRD) in Africa. Specifically, to develop a culture‐sensitive training framework for AD/ADRD caregivers, we explored AD/ADRD caregivers’ cultural embeddedness in managing people living with AD/ADRD (PLWAD/ADRD) in Ibadan, Nigeria.

**Method:**

Using hermeneutic phenomenological qualitative research design, we interviewed 23 caregivers providing care to PLWAD/ADRD in Ibadan, a Yoruba‐speaking ethnic group of Nigeria. Caregivers participated in in‐depth interviews (IDI) to share their experiences in managing AD/ADRD PBO. To capture the interpretive value of the caregivers’ experiences, we developed inductive and deductive codes and applied them to caregivers’ narratives using Nvivo 14. Based on the reflexivity of the multidisciplinary research team, themes, and sub‐themes about the cultural elements of caregivers' response to the PBO of PLWAD/ADRD were developed.

**Result:**

Among the 23 caregivers, 16 were female; five received a monthly salary; two were non‐members of the Yoruba ethnic group. We found five culturally sensitive themes, broadly categorized into cultural universals and specifics. The Yoruba‐specific aspects included (1) respect and filial piety, (2) non‐verbal cues, and (3) communal living arrangements and rotational care. Meanwhile, the cultural universals centered on (4) reciprocity/fear of retribution and (5) patience. Caregivers showed respect and filial piety by staying quiet, speaking in a low tone, minimizing correction, and refraining from commenting on PLWAD/ADRDs' self‐efficacy. Non‐verbal cues, like facial expressions, played a crucial role. The communal living arrangement proved valuable in managing negative behaviors like wandering. Reciprocity and retribution were embedded beliefs, and patience was a culturally universal approach requisite in the care of PLWAD/ADRD.

**Conclusion:**

The five cultural themes are important training areas for managing the behavioral and psychological outcomes of AD/ADRD. Mixed‐methods studies are required to explore the dimensions and generalizability of the five cultural constructs to strengthen Africa’s AD/ADRD caregiving workforce.

